# Cross-talk between cancer cells and their neighbors via miRNA in extracellular vesicles: an emerging player in cancer metastasis

**DOI:** 10.1186/s12929-019-0500-6

**Published:** 2019-01-12

**Authors:** Akiko Kogure, Nobuyoshi Kosaka, Takahiro Ochiya

**Affiliations:** 1 Division of Molecular and Cellular Medicine, National Cancer Center Research Institute, 5-1-1 Tsukiji, Chuo-ku, Tokyo, 104-0045 Japan; 2 Department of Translational Research for Extracellular Vesicles, Tokyo Medical University, 6-7-1 Shinjuku, Shinjuku-ku, Tokyo, 160-0023 Japan; 3 Institute of Medical Science, Tokyo Medical University, 6-7-1 Shinjuku, Shinjuku-ku, Tokyo, 160-0023 Japan

**Keywords:** Cancer metastasis, microRNA, Extracellular vesicle, Microenvironment

## Abstract

Cancer metastasis is the major cause of mortality in cancer cases and is responsible for cancer deaths. It is known that cancer cells communicate with surrounding microenvironmental cells, such as fibroblast cells, immune cells, and endothelial cells, to create a cancer microenvironment for their progression. Extracellular vesicles (EVs) are small vesicles that can be secreted by most types of cells and play an important role in cell-to-cell communications via transferring bioactive cargos, including variable RNAs, such as microRNAs (miRNAs), to recipient cells. miRNAs are a class of small noncoding RNAs that posttranscriptionally regulate gene expression. The transfer of them to recipient cells influences the metastatic process of primary tumors. In this review, we summarize the function of miRNAs packaged in EVs in cancer metastasis and discuss the clinical utility of miRNAs in EVs.

## Introduction

The most common of cancer-related deaths are due to metastasis of the primary tumor that develop years to decades after apparent cures [1, 2]. In facts, melanoma, breast, and prostate cancers can recur many years or even decades after seemingly effective treatment has ended because of its metastasis to different parts of the body [3]. The main steps in metastasis are local tumor cell invasion into stroma, detachment and circulation of tumor cells, extravasation into a secondary site, and angiogenesis at the metastatic site to survive [4, 5]. Some cancer cells are arrested and remain dormant for many years [3, 6–8]. Tumor dormancy is an adaptation to stress in order to survive in a hostile microenvironment, which is characterized as immune escape from the host immune systems, the balanced cell proliferation and apoptosis, non-angiogenic feature, cell cycle arrest, and chemotherapy resistance [3–5]. Therefore, the understanding the strategy of metastatic and dormant state cell survival is required for the prevention of cancer recurrence. For survival in the metastatic site, cancer cells interact with other cells in the metastatic site [2–4].

Extracellular vesicles (EVs), which consist of a double layered lipid membrane, are used as communication tools between cells. There are several types of EVs, such as apoptotic bodies, microvesicles and exosomes. They are usually differentiated by their mechanism of biogenesis and size [9–11]. Exosomes were seen as trash vesicles for the elimination of cellular components, however, Raposo et al. showed that exosomes have a role in communication between cells [12]. So far, many important biological functions of exosomes have been plausibly revealed including cancer in the recent years [13]. A number of studies suggest that cancer cells communicate with each other and with neighboring microenvironmental cells via exosomes containing oncogenic molecules in the process of metastasis [14–16]. The Paget’s “seed-and-soil theory” is concept of the premetastatic niche in which an environment in a secondary organ contributes to the metastasis of a primary tumor [17]. Primary metastatic cells deliver oncogenic molecules in exosomes, and this delivery creates a premetastatic niche in the target organ that leads to the metastasis.

**Fig. 1:**
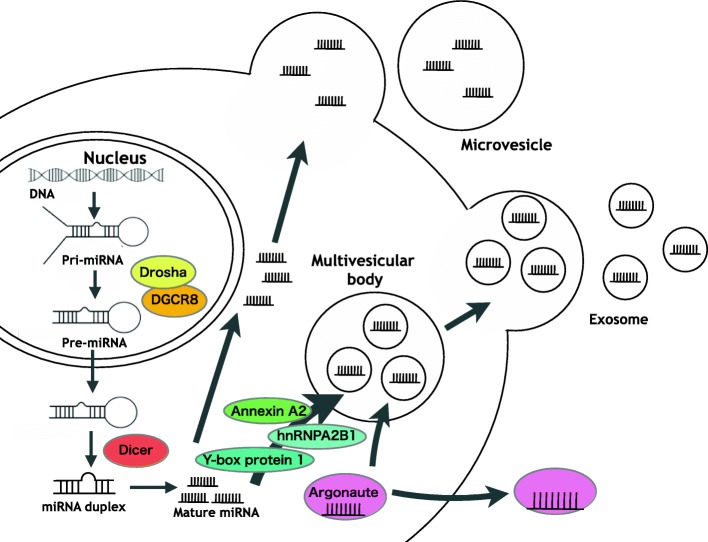
Schematic of miRNA biogenesis and release to the extracellular space. In the nucleus, miRNA genes are transcribed into pri-miRNAs and are processed to pre-miRNAs by Drosha and DGCR8. After further processing by Dicer, mature miRNAs form a complex (RISC) with Argonauts protein and other proteins, and are exocytic transferred with Ago2. In addition, miRNAs can be exported to the extracellular space with EVs. These miRNAs are packaged into EVs by proteins such as sumoylated hnRNPA2B1, Annexin A2, Y-box protein 1, and Ago2, and transferred to the circulation

A growing number of studies have already demonstrated that variable RNAs, such as microRNAs (miRNAs), long noncoding RNAs, and mRNAs, in exosomes can be transported between cells and have oncogenic or antioncogenic function in recipient cells. miRNAs are a class of small noncoding RNAs that posttranscriptionally regulate gene expression [18]. In the miRNA pathway, primary miRNA (pri-miRNAs) transcripts are cleaved by the microprocessor complex, which is composed of the ribonuclease III enzyme, Drosha, and its co-factor DiGeorge syndrome critical region gene 8 (DGCR8) [19, 20]. The processed products, termed precursor miRNAs (pre-miRNAs), are exported to the cytoplasm, where the pre-miRNA stem-loop is processed by another RNase III, Dicer, thus generating mature miRNAs. Mature miRNAs form the RNA-induced silencing complex (RISC) with Argonaute protein and other proteins, and then, the RISC recognizes and represses target gene expression (Fig. 1) [18–21]. It has been shown that deregulation of miRNA is tightly linked to cancer [22]. The difference of miRNA expression among cancer types has been well investigated by using comprehensive analyses [23, 24]. Moreover, it has also well-documented miRNAs contributing to oncogenesis or tumor suppression in many types of cancer [25, 26]. Thus, multiple roles of miRNAs have been reported in cancer progression cell autonomously.

miRNAs also act non-cell autonomously. Valadi et al. originally identified miRNAs inside of EVs from mast cell cultures and demonstrated that these miRNAs can be delivered to other cells [27], and they can function by transferring to recipient cells through EVs and regulate gene expression [28]. These miRNAs are sorted into EVs by the proteins such as sumoylated hnRNPA2B1, a heterogeneous nuclear riboprotein [29], Annexin A2 [30], Y-box protein 1 [31], and Ago2 [32] (Fig. 1). In some cases, the changes in miRNA sorting into EVs have been linked to tumor progression. A recent report has shown that major vault protein (MVP) regulated the sorting of tumor suppressive miRNA into EVs, which resulted in tumor progression [33]. To data, it is well known that miRNAs encapsulated in EVs have an important role in tumor progression through interaction between cancer cells and microenvironmental cells [34]. Moreover, circulating miRNAs have emerged as a potential biomarker for cancer diagnosis and prognosis [35–37]. In this review, we summarize the function of miRNAs in EVs for cancer metastasis. Then, we will discuss the potential applications for treatment strategies of metastatic cancer and recurrence.

## Cross-talk between tumor cells and endothelial cells via miRNAs in EVs

In metastasis, the recruitment of new blood vessels is essential for cancer cells. The process of angiogenesis is a dynamic and tightly regulated process among angiogenic factors, extracellular matrix components, and endothelial cells (EC) [38, 39]. Although various angiogenesis-related factors secreted by tumor cells have been reported, recent studies have shown the important role of EVs in the process of angiogenesis in primary tumor and distance metastatic sites [40]. Breast cancer cells can metastasize to areas far from the primary site, such as lymph nodes, bone marrow (BM), lung and liver. It was revealed that highly metastatic breast cancer cell lines expressed miR-210, which is also highly detected in their EVs. miR-210 in EVs was transferred to ECs and enhanced EC migration and capillary formation [41]. A current study showed that hepatocellular carcinoma cell-derived miR-210 also promotes EC migration and capillary formation. They showed the correlation between the high level of miR-210 in the serum from hepatocellular carcinoma patients and high microvessel density [42]. These data suggest that miR-210 in EVs has an important role in angiogenesis during tumor progression. Melanoma-derived EVs containing miR-9 also affect angiogenesis in distance metastatic sites. miR-9 in EVs is transferred to ECs, and it reduces the suppressor of cytokine signaling 5 (SOCS5) expression, an inhibitor of the JAK-STAT pathway in ECs [43]. This signaling cascade promotes EC migration and tumor angiogenesis. Some reports showed that miRNAs in EVs from cancer cells contributed to destroy the formation of tight junctions. EVs secreted from meningeal metastatic breast cancer cells contain miR-105, and they are transferred to ECs and suppress their tight junctions through downregulation of Zonula Occludens protein 1 (ZO-1), which is a tight junction protein between cells. The suppression of ZO-1 expression leads to the promotion of metastasis by destroying vascular endothelial barriers [44]. In addition, it was demonstrated that miR-181c in EVs derived from a brain metastatic breast cancer cell line induce the destruction of vascular endothelial barriers by targeting phosphoinositide-dependent protein kinase-1 (PDPK1), which leads to downregulation of cofilin phosphorylation [45]. These studies suggest that miRNAs secreted by metastatic breast cancer cells have regulatory roles in the destruction of tight junctions, resulting the promotion of brain metastasis. Taken together with the papers discussed above, the phenotype of endothelial is regulated by miRNAs in cancer-derived EVs in tumor progression and metastasis.

## Immune systems modulation via miRNAs in Cancer cell-derived EVs

Tumor cells escape from the anti-tumor immune response at the primary site and prepare the environment of the future metastatic site at a distant region [46]. During each step of the metastatic cascade, tumor cells are exposed to the immune system, which can recognize them and restrict their growth. Several groups have demonstrated that cancer cells and immune cells contact each other through miRNAs in EVs to induce immune reactions.

One such mechanism is involved in an increase in the population of regulatory T cells (Tregs), a subset of CD4+ T cells that plays an important role in maintaining self-tolerance and modulating immune responses [46, 47]. Since Tregs suppress the T cell-mediated immune response, tumor cells recruit Tregs to evade the host immune response [48]. Due to the importance of Tregs in tumor immune evasion, the depletion of Tregs is a potential therapy strategy for cancer. A report showed that the transfer of miRNA-214 in EVs derived from Lewis lung carcinoma to T cells downregulated PTEN and promoted Treg expansion [49]. Thus, miRNAs from tumor cells targeting immune cells and the immune system represent an active pathway for tumor immune evasion.

Macrophages are a major component of tumor-infiltrating immune cells and associate with tumor metastasis. It is known that interferon-γ (IFNγ) and Toll-like receptor (TLR) ligands activate macrophages to eliminate tumor cells. Fabbri et al. showed that miRNA in EVs directly activates TLRs. Lung cancer cells secrete substantial miR-21- and miR-29a-containing EVs, and these miRNAs function as ligands of TLRs in the surrounding immune cells [50]. This process results in the release of IL-6, TNF-α and other pro-inflammatory cytokines via a nuclear factor kappa B (NF-kB) pathway-mediated pro-inflammatory response, which makes the tumor microenvironment into a pro-metastatic niche. On the other hand, tumor-associated macrophages (TAMs) have the ability to promote tumor invasion and metastasis. TAMs are alternatively activated cells induced by IL-4-releasing CD4+ T cells. EVs containing high levels of miR-233 secreted by the TAMs can be transferred to breast cancer cells and promote their invasiveness by regulating the myocyte enhancer factor 2c (Mef2c)-β-catenin signaling pathway [51] . The reduction of Mef2c is related to nuclear accumulation of β-catenin to promote the invasiveness of the breast cancer cell lines [51]. A recent study showed that miR-203 from tumor cells could induce the differentiation of monocytes to M2 macrophages in vivo, which promotes distant metastasis [52].

EVs also inhibit immune responses by dendritic cells (DCs) and weaken anti-cancer immune processes by regulating the differentiation and maturation of DCs and their antigen-processing ability. A previous study revealed that pancreatic cancer-derived EVs attenuated DC-mediated tumor suppressive responses initiated by TLR4. They revealed that pancreatic cancer-derived EVs containing miR-203 regulated the expression of TLR-4 [53]. In addition to this study, they also found that pancreatic cancer-derived EVs containing miRNAs could be delivered to DCs, which could decrease the expression level of MHC II and induce the immune tolerance of DCs [54]. Thus, miRNAs in EVs from tumor cells modulate the immune system by increasing in the population of Tregs, activating macrophages and inducing immune tolerance of DCs.

## Communication by EV-derived miRNAs between tumor cells and fibroblasts

It is known that cancer cells communicate with fibroblast via EVs, and this leads to the progression of metastasis [55]. The fibroblasts in tumors are involved in epithelial-mesenchymal transition (EMT) and chemotherapy resistance by contacting with cancer cells and each other [56]. A report showed that pancreatic adenocarcinoma-secreted EVs were taken up by lymph node stromal cells and fibroblasts in the distant metastatic site. The tumor-secreted EVs were enriched in miR-494 and miR-542-3p and regulated the expression of cadherin-17, MAL and TRAF4 genes, leading to the upregulation of matrix metalloproteinases in recipient cells [57]. Tumor EVs also cause fibroblasts to differentiate into myofibroblasts, called cancer-associated fibroblasts (CAFs), inducing extracellular matrix remodeling and leading to tumor growth, invasion and metastasis [58]. It was reported that in the lung metastatic niche, high-metastatic hepatocellular carcinoma (HCC) cells exhibit a greater capacity to convert normal fibroblasts to CAFs than low-metastatic HCC cells [59]. The high-metastatic HCC cells secrete miR-1247-3p via EVs and activate β1-integrin-NF-κB signaling in normal fibroblasts. The activation of this signaling leads normal fibroblasts to become CAFs [59]. Moreover, high serum miR-1247-3p levels correlate with lung metastasis in HCC patients [59]. These results from the same group suggest that cell-cell communication between tumor cells and fibroblasts is mediated by tumor-derived EVs that control lung metastasis of HCC, providing potential targets for the prevention and treatment of cancer metastasis. On the other hand, fibroblast-derived EVs also contribute to cancer metastasis. Josson et al. revealed that miR-409 released from CAFs in prostate cancer were involved in prostate tumorigenesis by inducing EMT and downregulating the expression of the tumor suppressor genes, RSU1 and STAG2 [60]. Another group also showed that EMT was modulated by CAF-derived EVs containing miRs-21, −278e, and − 143, which influence breast cancer cell phenotype and aggressiveness [61]. Yeung et al. showed that miR-21 is transferred from CAFs to cancer cells, which suppressed apoptosis in ovarian cancer cells and induced chemo-resistance by binding to its direct target, APAF1 [62]. Moreover, a report showed that breast cancer-secreted miR-122 suppressed glucose uptake in lung fibroblasts by targeting pyruvate kinase. The increased glucose availability permitted metastatic breast cancer cells to adapt to their nutrient requirements and facilitated metastatic seeding [63]. These reports indicate that cancer cells and CAFs communicate with each other via miRNAs in EVs to maintain the metastatic niche.

## Dormancy induction via miRNAs in bone stroma cell-derived EVs

Challenges of treatment for cancer dormancy are important, since quiescent cancer cells exhibit chemotherapy resistance, with a high possibility of recurrence. It has been shown that communication via EVs from cancer metastatic niche to cancer cells can induce the dormancy state. A report showed that breast cancer cells that received EVs from bone stromal cells entered the G0 phase of the cell cycle. These EVs contained miR-127, − 197, − 222 and − 223, which are proliferation-inhibiting miRNAs [64]. Subsequent research has provided additional insight into cancer cell-stromal cell communication with EVs, plays a role in cell dormancy. They demonstrated that BM metastatic cancer cells that received BM-mesenchymal stem cell (MSC)-derived EVs exhibited dormancy. Moreover, the abundance of miR-23b was higher in the BM-metastasized cell-derived EVs than that in fibroblast-derived EVs, and cancer cells transfected with miR-23b showed a dormant phenotype due to the induction of suppression of cell cycling and mortality [65]. Another study also showed that BM-MSC-derived miRNAs transported by EVs also induced dormancy in breast cancer. They found that miR-222 and -223 were more effective in inducing dormancy in BM-MSCs that were primed with breast cancer cells compared to nonprimed BM-MSCs [66]. Furthermore, the effects were not observed using a low-metastatic line. These studies suggest that BM-stromal cells and BM-MSCs play an important role in inducing breast cancer cell dormancy and subsequent recurrence. Therefore, targeting molecules secreted through miRNAs from BM-stromal cell or BM-MSC may prevent or delay cancer recurrence.

## Function of miRNAs secreted via EVs in respond to environmental factors

Microenvironmental stress conditions, such as hypoxia and nutrient depletion, influence the survival of cancer cells and are related to the level of cancer metastasis. Although the regulation mechanism is still unclear, some reports have suggested that the miRNAs in EVs have functions in response to environmental conditions. A report showed that breast cancer cells under hypoxic conditions released pro-angiogenic EVs enriched in miR-210 [67]. Moreover, hypoxic hepatocellular.

Carcinoma also induces angiogenesis via miR-23a in EVs [68]. When the miR-23 in EVs was incubated with chick chorioallantoic membrane, higher blood vessel density and hemoglobin levels were observed. Cancer cells also develop strategies using miRNAs in EVs to increase their availability to glucose, such as angiogenesis to gain nutrients from blood. As mentioned before, miR-122 is abundantly released by breast cancer cells and can promote metastasis by adapting the metabolic environment into a premetastatic niche [63]. The inhibition of glucose uptake in the surrounding cells could lead to making a favorable environment for cancer cells. Thus, miRNAs are secreted in response to environmental stress and modulate the cancer metastatic niche.

## Perspectives and conclusions

**Fig. 2:**
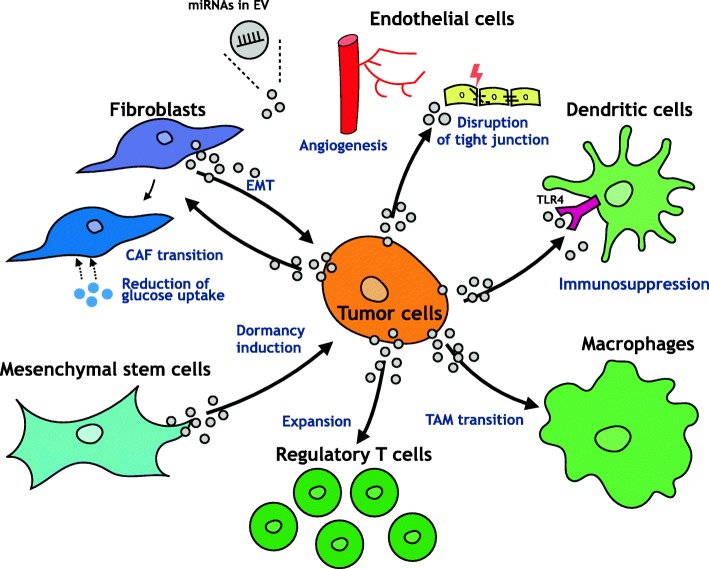
miRNA-mediated cross talk via EVs between cancer cells and environmental cells for tumor progression. It is known that tumor-secreted miRNAs transfer to environmental cells and function in the recipient cells. For instance, EVs mediate the delivery of miRNAs from cancer cells to ECs, resulting in the promotion of angiogenesis or the disruption of tight junctions. Moreover, tumor-derived miRNAs are transferred from cancer cells to immune cells, such as DCs and Tregs, and suppress the host immune system. In addition to this, tumor-derived miRNAs are transferred to macrophages and induce TAM transition, which promotes tumor progression. Furthermore, CAF transition is induced by tumor-derived miRNAs via EVs. Environmental cell-derived miRNAs are also transferred to cancer cells via EVs. Mesenchymal stem cell-derived miRNAs are transferred to tumor cells through EVs and induce tumor dormancy. In addition, fibroblast-derived miRNAs in EVs are transferred to tumor cells and induce EMT

Metastasis is a final and fatal step in the progression of solid tumors [5]. As mentioned in this review, dozens of studies have shown that miRNAs encapsulated in EVs have an important role in the process of cancer metastasis through direct contact between cancer cells and environmental cells, such as endothelial cells, immune cells, and stromal cells (Table 1 and Fig. 2). Moreover, the secretion profile of miRNAs is changed in response to environmental stress. These findings suggest that secreted miRNAs from cancer cells or environmental cells could reflect the tumor progression level. It is known that secreted miRNAs are detected in a variety of bodily fluids, such as blood, tears and urine [35, 69]. This detection suggests that miRNAs in EVs are a promising strategy for identifying specific biomarkers for the diagnosis and prognosis of cancer metastasis.

**Table 1:** Function of miRNAs in EVs in cancer metastasis

Phenotype	Donor Cells	Recipient Cells	miRNA	Target Gene of miRNA	Reference
Induction of angiogenesis	Breast cancer cell		miR-210	–	[41]
	HCC cell	EC	miR-210	SMAD4 and STAT6	[42]
	Melanoma cell		miR-9	SOCS5	[43]
Destruction of endothelial barriers	Breast cancer cell	EC	miR-105	ZO-1	[44]
Breakdown of blood-brain barrier	Breast cancer cell	EC	miR-181c	PDPK1	[45]
Treg expansion	Lewis lung carcinoma	CD4 + T cell	miR-214	PTEN	[49]
Promotion of inflammation	Lung cancer cell	Macrophage	miR-21 and -29a	TLR	[50]
Promoting invasion	TAM	Breast cancer cell	miR-233	Mef2s	[51]
TAM transition	Colorectal cancer	Monocyte	miR-203	–	[52]
Dysfunction of DC	Pancreatic cancer	DC	miR-203	TLR4	[53]
Immune tolerance of DC	Pancreatic cancer	DC	miR-212	RFXAP	[54]
Up-regulation matrix metalloproteinases	Pancreatic adenocarcinoma	Fibroblast	miR-494 and − 542-3p	Cadherin-17, MAL and TRAF4	[57]
CAF transition	HCC cell	Fibroblast	miR-1247-3p	B4GALT3	[59]
Induction of EMT	CAF	Prostate cancer cell	miR-409	RSU1 and STAG2	[60]
		Breast cancer cell	miR-21, −278e, and − 143	–	[61]
Inhibition of apoptosis and induction of drug resistance	CAF	Ovarian cancer cell	miR-21	APAF1	[62]
Reduction of glucose uptake	Breast cancer cell	Fibroblast	miR-122	Pyruvate kinase	[63]
Suppression of proliferation	Bone stromal cell	Breast cancer cell	miR-127, −197, −222 and − 223	CXCL12	[64]
Induction of dormancy	BM-MSC	Breast cancer cell	miR-23b	MARCKS	[65]
Induction of dormancy and drug resistance	BM-MSC	Breast cancer cell	miR-222 and − 223	–	[66]

Foot Note: *miRNA* microRNA, *HCC* hepatocellular carcinoma, *TAM* tumor-associated macrophage, *CAF* cancer-associated fibroblast, *BM* bone marrow, *MSC* mesenchymal stem cell, *EC* endothelial cells, *DC* dendritic cell, *SOCS5* suppressor of cytokine signaling 5, *ZO-1* zonula occludens protein 1, *PDPK1* phosphoinositide-dependent protein kinase-1, *PTEN* phosphatase and tensin homolog, *TLR* Toll-like receptor, *RFXAP* regulatory factor X-associated protein, *Mef2c* myocyte enhancer factor 2c, *Treg* regulatory T cell

One important issue for cancer therapy is recurrence after long periods of treatment. As we mentioned in the Introduction, understanding the strategy of dormant state cell survival is necessary for prevention of cancer recurrence, since some metastasized cancer cells are arrested and remain dormant for many years [3, 6–8]. Currently, several studies have revealed that miRNAs have functions via EVs in entering dormant state [64–66]. If these miRNAs can be detected before cancer relapse, it might be possible to find metastasized cancer cells and prevent cancer recurrence in its early stages. Moreover, if the transfer of miRNAs, which creates a niche that harbors dormant tumor cells, could be reduced, this reduction would effectively inhibit cancer metastasis and help prevent cancer recurrence.

Thus, the miRNAs in EVs derived from cancer cells and environmental cells can be used as a biomarker for cancer metastasis and as a target for cancer therapy.

## Abbreviations

BM: Bone marrow
CAF: Cancer-associated fibroblast
DC: Dendritic cell
DGCR8: DiGeorge syndrome critical region gene 8
EC: Endothelial cells
EMT: Epithelial-mesenchymal transition
EV: Extracellular vesicle
HCC: Hepatocellular carcinoma
IFNγ: Interferon-γ
Mef2c: Myocyte enhancer factor 2c
miRNA: MicroRNA
MSC: Mesenchymal stem cell.
MVP: Major vault protein
NF-κB: Nuclear factor kappa B
PDPK1: Phosphoinositide-dependent protein kinase-1
pre-miRNA: precursor miRNA
pri-miRNA: primary miRNA
PTEN: Phosphatase and tensin homolog
RISC: RNA-induced silencing complex
SOCS5: Suppressor of cytokine signaling 5
TAM: Tumor-associated macrophage
TLR: Toll-like receptor
Treg: Regulatory T cell
ZO-1: Zonula occludens protein 1
